# Evaluation of the effect of etamsylate on thromboelastographic traces of canine blood with and without the addition of heparin

**DOI:** 10.1080/01652176.2023.2260449

**Published:** 2023-09-16

**Authors:** Vicente Herrería-Bustillo, Maite Masiá-Castillo, Helen R. P. Phillips, Laura Gil-Vicente

**Affiliations:** a Escuela de Doctorado. Facultad de Veterinaria y Ciencias Experimentales, Universidad Católica de Valencia San Vicente Mártir, Valencia, Spain; b Department of Veterinary Medicine and Surgery, Universidad Católica de Valencia San Vicente Mártir, Valencia, Spain; c Department of Terrestrial Ecology, Netherlands Institute of Ecology (NIOO-KNAW), Wageningen, the Netherlands; d Department of Environmental Science, Saint Mary’s University, Halifax, Canada

**Keywords:** Dicynone. 2, 5-dihydroxy-benzene-sulfonate diethylammonium salt. Heparin. Thromboelastography. Reversal of anticoagulation. Fibrinolysis

## Abstract

The objective of this study was to investigate the effect of etamsylate on canine blood and heparinised canine blood from healthy dogs using thromboelastography (TEG). Citrated blood was obtained from twenty healthy client-owned dogs, and 3 experiments were performed. Experiment 1 compared TEG in blood versus blood with etamsylate (250 mM). Experiment 2 evaluated TEG in heparinised blood (1 U/mL) with and without the addition of etamsylate (250 mM). Experiment 3 evaluated dose escalation of etamsylate (control, 250 μM, 500 μM and 1000 μM) in heparinised blood (1 U/mL). The addition of etamsylate to canine blood in experiment 1 increased the percentage of clot lysis at 30 min (z = −2.103, *p* = .035) and 60 min (z = −1.988, *p* = .047), suggesting that etamsylate could have a fibrinolytic effect. When etamsylate was added to heparinised canine blood (1 U/mL), etamsylate produced a dose-dependent inhibition of the effect of heparin when higher concentrations of etamsylate were used (500 μM and 1000 μM). The linear mixed effects model showed significant increases in α angle and maximal amplitude when high dose etamsylate was added compared to the control. In conclusion, etamsylate could be used at higher doses to inhibit the effect of heparin in dogs when protamine might not be available. However, etamsylate might have a fibrinolytic effect when used in healthy dogs.

## Introduction

Etamsylate (also called 2,5-dihydroxy-benzene-sulfonate diethylammonium salt or Dicynone) is a haemostatic drug used clinically to stop bleeding in animals and humans. Its mechanism of action at the haemostatic level is complex. Etamsylate reduces the synthesis of prostacyclin, thus promoting platelet aggregation (Garay et al. 2006). Etamsylate also promotes platelet aggregation by stimulating the expression of p-selectin, a cell adhesion protein (Alvarez-Guerra et al. 2002; Hernandez et al. 2004; Garay et al. 2006; Segura et al. 2007). A recent in-vitro study has shown the ability of etamsylate to inhibit the anticoagulant effect of heparin in canine blood samples by normalising activated partial thromboplastin time (aPTT) (Cobo-Nuñez et al. 2018). Etamsylate is a drug with very low toxicity and a wide margin of tolerance, as it is not metabolised and is rapidly eliminated *via* the kidneys as unchanged etamsylate. In dogs and cats, good tolerance has been observed at doses up to 200 mg/kg (Garay et al. 2006). Clinically recommended dosages in dogs and cats are in the range are in the range of 6.25 to 12.5 mg/kg.

Etamsylate has been used clinically in dogs, cats, horses, cattle, and humans with bleeding disorders, although the number of clinical publications in veterinary medicine is limited, with only one publication. The only veterinary publication available of the clinical use of etamsylate described a reduction in hemolactia in lactating dairy cows when etamsylate was administered at 15 mg/kg daily for three consecutive days (Fraile et al. 2019).

Most of the human literature has focused on severe menstrual bleeding, surgical bleeding and periventricular bleeding in preterm infants. A systematic review that evaluated seven trials enrolling 1410 preterm infants found that, although infants treated with etamsylate had significantly less intraventricular haemorrhage than controls at <31 weeks and <35 weeks of gestation, there was no significant difference detected in neonatal mortality or neurodevelopmental outcome (Hunt and Hey 2010). Another systematic review that evaluated 1770 women with heavy menstrual bleeding did not find a reliable decrease in menstrual blood loss when etamsylate was used (Bofill Rodriguez et al. 2022). A recent study evaluated the use of a combination of etamsylate with tranexamic acid (TXA) in children undergoing cardiac surgery under cardiopulmonary bypass (CPB). The authors found that patients receiving the etamsylate-TXA combination had less blood loss and required fewer transfusions than patients receiving TXA or in the control group (El Baser et al. 2021). Interestingly, heparin had been used as anticoagulant during CPB, so etamsylate might have helped inhibit residual heparin action.

Thromboelastography (TEG) is a laboratory analysis technique that evaluates the viscoelastic properties of whole blood coagulation under low shear stress conditions (McMichael and Smith 2011). Unlike traditional haemostasis tests, it provides global information on haemostatic function from the beginning of blood clot formation until its destruction by fibrinolysis. The thromboelastograph provides various measurements that evaluate the functionality of the enzymatic part of coagulation (R time), fibrinogen [K, α angle, maximal amplitude (MA)], platelets (MA) and fibrinolysis [percentage of clot lysis at 30 (LY30) and 60 min (LY60)] in an integrated, global way. Another variable representing shear elastic modulus, global clot strength (G), is calculated as *G* = 5000 × MA/(100 − MA) (McMichael and Smith 2011).

The objective of our study was to evaluate the effect of etamsylate on canine blood and heparinised canine blood using TEG. Our hypothesis was that etamsylate would reverse the effects of heparin and would produce hypercoagulability in canine blood.

## Material and methods

Twenty healthy client-owned dogs of various breeds over one year of age and over 10 kg were recruited to participate in the study. The study protocol was reviewed and approved by the Committee on Ethics in Animal Experimentation code CEEAUCV2005. Dog owners were asked to sign a written informed consent prior to participating in the research. To verify the health status of the dogs, a complete physical examination, haematology, serum biochemistry, prothrombin time (PT) and activated partial thromboplastin time (aPTT) were performed. Subsequently, 10 mL of blood were extracted from a previously unused jugular vein using a 10 mL syringe and 21 Ga needle and the blood was stored in 3.2% trisodium citrate tubes. Unused citrated blood was centrifuged, and plasma was separated and frozen at −80 °C for future studies. Samples were kept at room temperature for 30–60 min prior to analysis (Flatland et al. 2014). Etamsylate 125 mg/mL (Hemo 141, Ecuphar, Spain) was diluted in sterile water to obtain concentrations of 5 mM, 10 mM and 20 m. A potassium phosphate buffer solution (PBS, pH 7.4) was used for all samples to be equally diluted whether or not etamsylate was added, as well as independently of the concentration used (Fletcher et al. 2014).

The current study included three different experiments. Experiments 1 and 2 were performed simultaneously with blood from 10 healthy dogs. Experiment 1 consisted of comparing TEG values in blood samples from 10 healthy dogs with and without the addition of etamsylate to evaluate the effect of this drug on viscoelastic testing. The first sample was created by mixing 900 μL of citrated blood and 100 μL of PBS. The second sample was produced by mixing 900 μL of citrated blood, 50 μL of PBS and 50 μL of etamsylate 5 mM to achieve a concentration of etamsylate of 250 μM. The concentration of etamsylate was calculated based on its volume of distribution in humans (0.21 L/kg) (Homedes 2002) and a standard IV dose of 12.5 mg/kg IV, assuming a single-compartment model using the formula: concentration = dose/volume of distribution. This concentration of etamsylate inhibited heparin action in canine blood in an in-vitro study (Cobo-Nuñez et al. 2018). In experiment 2, we compared TEG values in heparinised blood with and without the addition of etamsylate, using blood from the same dogs from experiment 1. Commercial 1% unfractionated heparin (1000 U/mL) (Heparina sódica Sala, Reig Jofre, Barcelona, Spain) was diluted to 0.1% heparin (100 U/mL) in sterile water. The first sample was prepared as follows: 890 μL of citrated blood, 10 μL of 0.1% heparin and 100 μL of PBS; and the second sample: 890 μL of citrated blood, 10 μL of 0.1% heparin, 50 μL of 5 mM etamsylate and 50 μL of PBS to achieve a concentration of etamsylate of 250 μM. The concentration of heparin in each cuvette was 1 U/mL. This concentration has been estimated based on an intravenous loading dose of unfractionated heparin of 50 U/kg and its approximate volume of distribution (0.05 L/kg) (Diquélou et al. 2005), assuming a single-compartment model. Experiment 3 evaluated the effect of various etamsylate doses on canine heparinised blood. Three different etamsylate dilutions were used, 5, 10, and 20 mM, to achieve concentrations of 250 μM, 500 μM and 1000 μM in each cuvette, respectively. Four samples were created for experiment 3. The first sample contained 890 μL of citrated blood, 10 μL of 0.1% heparin and 100 μL of PBS; the second sample contained 890 μL of citrated blood, 10 μL of 0.1% heparin, 50 μL of 5 mM etamsylate and 50 μL of PBS to achieve a concentration of etamsylate of 250 μM; the third sample contained 890 μL of citrated blood, 10 μL of 0.1% heparin, 50 μL of 10 mM etamsylate and 50 μL of PBS to achieve a concentration of etamsylate of 500 μM; and the fourth sample contained 890 μL of citrated blood, 10 μL of 0.1% heparin, 50 μL of 20 mM etamsylate and 50 μL of PBS to achieve a concentration of etamsylate of 1000 μM. All samples were mixed manually by inverting the tubes 5 times and were incubated at room temperature for 5 min prior to performing TEG.

### Thromboelastography

All analyses were performed using a viscoelastic coagulation analyser (TEG 5000, Haemonics, USA). Following incubation, each 1 mL sample was added to a kaolin tube for activation. Tubes were inverted 5 times following the manufacturer’s instructions, and 340 μL of blood followed by 20 μL of calcium chloride were pipetted into the cuvettes, and the measurements were started. The variables obtained were reaction time (R), α angle, K, MA, G, LY30 and LY60.

### Statistical analysis

Normality of the data was assessed with the Shapiro-Wilk test. Continuous data were reported as mean ± standard deviation (SD) and median and interquartile range (IQR) when not normally distributed. Categorical data were reported as numbers and percentages. Variables from experiments 1 and 2 were compared using paired Student’s t-test for normally distributed data or Wilcoxon Signed-Rank for non-parametric data. Variables from experiment 3 were analysed with a mixed effects linear model using each of the TEG variables (R, K, α angle, MA, G and LY60) as response variables, sample type as the predictor variable and patient as the intercept-only random effect (to account for multiple samples from the same dog). LY30 was not included as a predictor model due to too low cardinality in the variable. Statistical significance was set at *p* < .05. The statistical analysis was carried out using two commercially available computer programmes [IBM SPSS version 26, New York, United States; and R Core Team (2021)].

## Results

Mean age of the dogs was 4.9 ± 2.9 years and median body weight was 25.9 Kg (IQR 8.8). There were 11 (55%) males and 9 (45%) females. Eight (40%) dogs were entire, and 12 (60%) were neutered. There were 7 crossbreeds, 5 Labrador, 2 Pointer, 2 Weimaraner and one each of the following: Belgian shepherd, German shepherd, Golden retriever, and Spanish waterdog.

**Figure 1.**
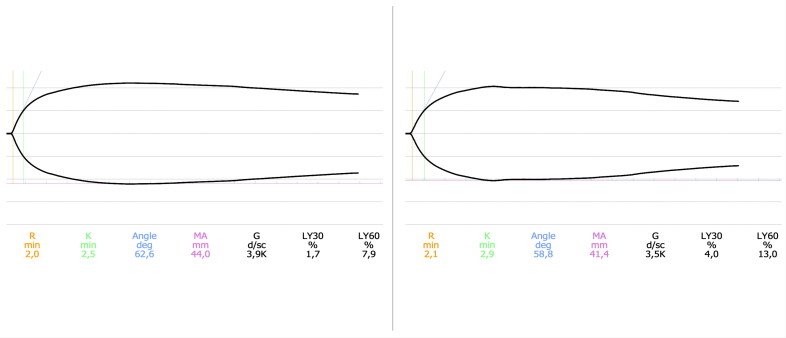
Graphical representation of a TEG performed on the blood of a healthy dog (left) and on the same patient following the addition of etamsylate 250 μM (right). The most significant finding is an increase in the percentage of clot lysis at 30 min (LY30) and 60 min (LY60).

Experiment 1 results are described in Table 1. The only variables that were significantly different following the addition of etamsylate were LY30 and LY60. The percentage of clot lysis increased at 30 min (z = −2.103, *p* = .035) and 60 min (z = −1.988, *p* = .047) post MA after adding etamsylate, suggesting that etamsylate could have a mild fibrinolytic effect (Figure 1).

**Table 1.** Comparison of TEG values of paired blood samples: control and with the addition of etamsylate 250 μM.

Variable	Control Mean ± SD or median (IQR)	Etamsylate 250 μM Mean ± SD or median (IQR)	Reference Intervals	*p* Value
R	2.5 ± 1.2	2.6 ± 0.9	4–9	.625
K	2.5 ± 1.0	2.9 ± 0.9	1–3	.433
α angle	64.1 ± 7.1	62.5 ± 6.2	59–74	.417
MA	50.2 ± 5.1	48.4 ± 6.2	55–74	.074
G	5133 ± 1085	4817 ± 1235	5300–13,200	.089
LY30	0.1 (0.6)	0.7 (2.2)	0–3	**.035**
LY60	3.5 (2.4)	4.7 (6.0)	0–15	**.047**

*Note:* TEG: thromboelastography; SD: standard deviation; IQR: interquartile range; R: reaction time; K: time to reach an amplitude of 20 mm after R; MA: maximum amplitude; G: global clot strength; LY30: percentage of clot lysis 30 min post MA; LY60: percentage of clot lysis 60 min post-MA. Significance was set a *p*<0.05

Results obtained from experiment 2 are depicted in Table 2. Thromboelastographic variables in heparinised blood and heparinised blood with the addition of etamsylate at a concentration of 250 μM were not significantly different between the two samples.

**Table 2.** Comparison of TEG values of paired heparinised blood samples (1 U/mL) without and with the addition of etamsylate 250 μM.

Variable	Heparin 1 U/mL Mean ± SD or median (IQR)	Heparin 1 U/mL + etamsylate 250 μM Mean ± SD or median (IQR)	*p* Value
R	6.9 ± 1.9	7.9 ± 2.4	.071
K	5.1 ± 3.9	4.7 ± 2.9	.234
α angle	42.3 ± 13.9	40.5 ± 12.4	.451
MA	39.9 ± 9.4	42.9 ± 7.2	.109
G	3506 ± 1396	3887 ± 1212	.192
LY30	0.1 (1.6)	0 (2)	.686
LY60	1.3 (5.8)	1.2 (7.9)	1.000

*Note:* TEG: thromboelastography; SD: standard deviation; IQR: interquartile range; R: reaction time; K: time to reach an amplitude of 20 mm after R; MA: maximum amplitude; G: global clot strength; LY30: percentage of clot lysis 30 min post MA; LY60: percentage of clot lysis 60 min post MA.

The linear mixed effects model performed on data from experiment 3 showed significant increases in α angle, MA and G compared to the control when using higher concentrations of etamsylate (500 μM and 1000 μM). When etamsylate was used at 500 μM, MA was estimated to be 52 mm vs 48.1 mm for the control (*p* < .001) and α angle 49.3° compared to 46.6° for the control (*p* = .01). When etamsylate was used at 1000 μM, MA was 52.7 mm vs 48.1 mm for the control (*p* < .001), and α angle was 51° vs 46.6° for the control (*p* = .01) (Figure 1). There was no significant difference between the estimates of MA, G or α angle between an etamsylate dose of 500 μM and 1000 μM. The remaining TEG values (R time, K and LY60) did not show significant changes at the various etamsylate doses (Table 3).

**Table 3.** TEG values obtained following the addition of Incremental doses of etamsylate to heparinised blood (1 U/mL).

Variable	Control Mean ± SD or median (IQR)	Etamsylate 250 μM Mean ± SD or median (IQR)	Etamsylate 500 μM Mean ± SD or median (IQR)	Etamsylate 1000 μM Mean ± SD or median (IQR)
R	7.6 ± 2.0	7.3 ± 2.5	7.3 ± 2.7	7.2 ± 1.8
K	3.9 ± 1.6	4.0 ± 1.4	3.5 ± 1.3	3.2 ± 0.8
α angle	46.6 ± 9	46.3 ± 10.6	49.3 ± 10	51 ± 7.2
MA	48.1 ± 8	48 ± 9.1	52 ± 8	52.7 ± 6.4
G	4837 ± 1505	4894 ± 1778	5679 ± 1821	5744 ± 1532
LY30	0 (0)	0 (0.3)	0 (0)	0 (0)
LY60	0.8 (2.7)	0.3 (5.5)	0.6 (2.6)	0.8 (2.7)

*Note:* TEG: thromboelastography; SD: standard deviation; IQR: interquartile range; R: reaction time; K: time to reach an amplitude of 20 mm after R; MA: maximum amplitude; G: global clot strength; LY30: percentage of clot lysis 30 min post MA; LY60: percentage of clot lysis 60 min post MA.

## Discussion

The addition of etamsylate to canine blood in the first experiment of this study did not produce any prothrombotic effects but paradoxically showed a mild hyperfibrinolytic effect (Figure 1). Although there is no consensus regarding the diagnosis of hyperfibrinolysis based on TEG in veterinary medicine, most references consider hyperfibrinolysis when LY30 ≥ 3% and LY60 ≥ 15% (Kelley et al., 2015; Fry et al. 2017). The hyperfibrinolytic effect noted was mild and probably has low importance. Moreover, the significance of these results should be interpreted with caution due to the low number of samples performed. Further studies that evaluate fibrinolysis in patients receiving etamsylate would be needed to confirm this finding. A tissue plasminogen-activated TEG might be required (Spodsberg et al. 2013). Although most drugs with fibrinolytic activity are limited to indirect and direct derivates of plasmin (Marder and Novokhatny 2010), with the development of novel diagnostic techniques to assess fibrinolysis, new drugs with fibrinolytic activity are being discovered (Carter et al. 2018). This is the first study to report hyperfibrinolysis following the use of etamsylate. To our knowledge, only one previous human study evaluated fibrinolysis in people treated with etamsylate. This study did not find a significant change in type I tissue plasminogen activator inhibitor, a marker of fibrinolysis in people with Von Willebrand disease following treatment with etamsylate (Hutton et al. 1988).

It is thought that etamsylate enhances coagulation due to increased expression of p-selectin (Alvarez-Guerra et al. 2002; Hernandez et al. 2004; Garay et al. 2006; Segura et al. 2007). P-selectin is a cell adhesion molecule expressed in the alfa granules of platelets, the Weibel–Palade bodies of endothelial cells and is also present in a soluble form in plasma. P-selectin is translocated to the surface of activated platelets during thrombus growth and promotes the recruitment of circulating leucocytes into the thrombus. P-selectin induces tissue factor (TF) expression in monocytes, further promoting thrombus growth. It is also thought that P-selectin enhances platelet-platelet interactions through the GPIba-IX-V complex (André 2004). Although TEG is able to document increased platelet function in dogs (Majoy et al. 2015), there are other techniques, such as impedance platelet aggregometry or flow cytometry, that focus specifically on platelet function. Several platelet function tests have been evaluated in veterinary medicine that could improve our understanding of etamsylate’s procoagulant actions (Christopherson et al. 2012).

The inhibition of the anticoagulant effect of heparin by etamsylate was first suspected following the discovery of the inhibition of vascular endothelial growth factors (VEGF) by its active anion dobesilate (2,5-dihydroxybenzene sulfonate) (Angulo et al. 2011; Njau et al. 2020). Vascular endothelial growth factor and fibroblast growth factor (FGF) are signalling proteins that stimulate angiogenesis. Both VEGFs and FGFs contain a heparin-binding domain (HBD). Heparin and heparan sulphates positively regulate the activity of VEGF. The blockade of these HBD on VEGF and FGF are therapeutic targets to inhibit angiogenesis in several human diseases such as neoplasia, diabetic retinopathy, rosacea or psoriasis (Angulo et al. 2011).

**Figure 2.**
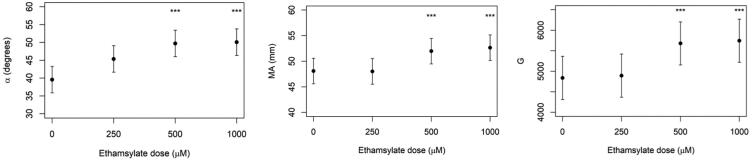
TEG variables MA, α angle and G obtained from adding Increasing doses of etamsylate to heparinised canine blood (1 U/mL). There are statistically significant increases in MA, α angle and G after adding etamsylate at 500 and 1000 μM, indicating partial reversal of anticoagulation. TEG: thromboelastography; MA: maximum amplitude. ***= statistically significant estimate.

The mixed effects model performed on data from experiment 3 showed significant increases in α angle, MA and G compared to the control (Figure 2) when using higher concentrations of etamsylate (500 μM and 1000 μM). However, a concentration of 250 μM did not produce significant changes in any of the parameters evaluated. A previous study found that etamsylate 100 μM and 250 μM normalised aPTT in canine blood treated with 0.2 U/mL of heparin (Cobo-Nuñez et al. 2018). The results of the present study agree with Cobo-Nuñez’s (2018) study, suggesting that when higher doses of heparin have been administered, a higher dose of etamsylate would be required to inactivate its anticoagulation activity. In such scenarios, the usual etamsylate dose of 6.25 to 12.5 mg/kg is likely to be ineffective and would have to be increased. Etamsylate has been used in dogs and cats up to 200 mg/kg with no appreciable side effects (Garay et al. 2006). Further studies are required to evaluate the appropriate dose of etamsylate depending on the dose of heparin administered. Protamine, the traditional reversal agent of heparin, acts in a similar way as the dose of protamine must be titrated based on the initial dose of heparin administered or using other techniques such as the activated clotting time (ACT) monitor (Hecht et al. 2020).

Although there are no studies comparing the effect of etamsylate versus protamine or heparinase to revert the anticoagulant effect of heparin, protamine and heparinase are probably more effective for this purpose. In the present study, etamsylate was able only to significantly change the variables MA and alfa angle compared to the control group, while R time was not significantly different. Both protamine and heparinase almost completely reversed the TEG variables R time, K and alfa angle in an in-vitro study of human blood treated with heparin 0.2 U/mL (Zmuda et al. 2000). Based on these results, further studies comparing the effects of these three drugs to reverse the anticoagulant action of heparin are warranted. Etamsylate might be a promising drug to reverse heparin action due to its more favourable safety profile. Protamine has several significant side effects, such as hypotension, inhibition of platelet aggregation, enhanced fibrinolysis and anaphylaxis (Hecht et al. 2020). Etamsylate might be used as an alternative to protamine to reverse the anticoagulant effect of heparin if the patient develops significant side effects from protamine or if protamine cannot be sourced.

Other published studies have evaluated the effect of unfractionated heparin on TEG tracings. These studies showed a greater prolongation of R in comparison with our study. One of the studies evaluated TEG 1 h after the administration of 200 U/kg SQ heparin (Pittman et al. 2010), whereas the other study administered doses between 200 and 300 U/kg and performed TEG 3 h after the administration (McLaughlin et al. 2017). The first study found progressive changes in the TEG tracing with a maximal change 3–5 h after dosing (Pittman et al. 2010). It is likely that R in the present study was lower because we used a lower heparin dose (equivalent to 50 U/kg IV) and performed TEG 5 min after mixing the blood with heparin.

The main limitation of our study is the small sample size. Another limitation is that TEG cannot account for the effect that etamsylate might have on vascular tone *via* prostacyclin inhibition. Moreover, all the experiments described in this study have used blood from healthy patients. It would be interesting to perform further studies using TEG in patients with haemostatic abnormalities treated with etamsylate.

## Conclusions

Etamsylate produced a dose-dependent partial inhibition of the anticoagulant effect of heparin in canine blood assessed by TEG. A mild fibrinolytic effect was observed when etamsylate was added to canine blood.
